# Learning new words via reading: The influence of emotional narrative context on learning novel adjectives

**DOI:** 10.1177/17470218241308221

**Published:** 2024-12-26

**Authors:** Yuzhen Dong, Matthew HC Mak, Robert Hepach, Kate Nation

**Affiliations:** 1 Department of Experimental Psychology, University of Oxford, Oxford, UK; 2 Department of Psychology, The University of Warwick, Coventry, UK

**Keywords:** Embodied cognition, emotion, word learning, reading, narratives

## Abstract

People learn new words in narrative contexts, but little is known about how the emotional valence of the narrative influences word learning. In a pre-registered experiment, 76 English-speaking adults read 30 novel adjectives embedded in 60 short narratives (20 positive, 20 negative, and 20 neutral valence). Both immediately after and 24 hr later, participants completed a series of post-tests, including speeded recognition, sentence completion, meaning generation, and valence judgement. Results showed that participants learned both the novel word form and its meaning. Compared with novel words experienced in the neutral contexts, those read in the emotional contexts (both positive and negative) showed better learning of orthographic form in the immediate post-test, but only those read in the negative context were recognised with greater accuracy in the delayed post-test. Furthermore, the valence of the context was reflected in the word meanings participants generated for each novel word, suggesting that word valence can be inferred from the valence of the contexts. Results from sentence completion and valence judgement were mixed, depending on the task demands. These findings are discussed with reference to theories of affective embodiment and the implications for learning abstract words are considered.

## Introduction

In language, emotional valence refers to the pleasantness of a word and the extent of its positivity or negativity (Warriner et al., 2013). This broadens the definition of emotion words from just describing an emotional state, such as *happy* or *sad*, to all content words. Word valence influences how early and how well a word is learnt (e.g., Kousta et al., 2011; Ponari et al., 2020); it also influences lexical processing in adults (e.g., Vinson et al., 2014). Most existing research relies on valence norms in which people rate the positivity of familiar words (e.g., Warriner et al., 2013). For an unfamiliar novel word, however, how do people learn its valence? One possibility is from the valence of the context in which it appears. In line with this, Snefjella and Kuperman (2016) reported a positive correlation between word valence and contextual valence, defined as the aggregate valence of the five content words immediately before and immediately after the word in text samples taken from a large corpus of email newsgroup postings. Word-learning experiments (e.g., Snefjella et al., 2020) have also investigated how valence of the word might be learned from emotional contexts, but the results have been mixed. In this pre-registered study, we investigated whether and how variations in the valence of emotional narrative context might influence the learning of new adjectives.

Word valence is known to influence lexical processing. In lexical decision for example, emotional words, whether positive or negative, are processed faster and with greater accuracy than neutral words, a phenomenon that persists regardless of the mode of word presentation (Kousta et al., 2009; Ponari et al., 2018; Scott et al., 2009, 2012). Further support comes from neuroimaging and electrophysiological studies, which demonstrate that words with more extreme valence elicit distinct neural responses compared with neutral words (Pauligk et al., 2019; Vigliocco et al., 2014; Yao et al., 2016). This processing advantage extends beyond isolated words to those presented within sentences, affecting both lexical processing and memory (Bayer et al., 2010; Scott et al., 2012).

Emotional valence predicts age-of-acquisition ratings and emotionally valenced abstract words tend to be earlier acquired than neutral ones (Kousta et al., 2011). Ponari et al. (2018) suggested that emotional valence provides a bootstrapping mechanism for acquiring abstract concepts. However, this valence effect is not uniformly observed across all age groups but is particularly pronounced in children aged 8 to 9 years (Lund et al., 2019; Ponari et al., 2018). Moreover, valence also impacts children’s learning and memory for newly taught abstract words, with emotionally valenced words being learned better and defined more accurately than neutral words (Kim et al., 2020; Ponari et al., 2020). These findings align with the affective embodiment account which proposes that emotional content aids in grounding abstract word meanings in emotional experiences, providing a motivational relevance that heightens processing efficiency (Vigliocco et al., 2014). However, although this account suggests that emotional valence facilitates the acquisition of abstract words, it does not have clear predictions about its directionality, i.e., whether positive valence or negative valence better supports word learning.

There are mixed findings as to the directionality of any valence influence on word processing and learning. Some studies have found a positivity advantage (e.g., Kuperman et al., 2014; Ponari et al., 2018; Yao et al., 2016), which can be explained by the Pollyanna principle, a global tendency for humans to remember pleasant things more accurately (Matlin & Stang, 1978). Unkelbach et al. (2008) proposed the informational density hypothesis, where positive information, being more elaborated and densely clustered, is processed faster than negative information. The greater interconnectivity of positive words in a denser semantic network might result in it being activated faster during word processing. Other studies, however, have also found a negativity advantage (e.g., Estes & Verges, 2008). This has been explained in terms of an increased vigilance for negative stimuli (Pratto & John, 1991) by which individuals have an intrinsic tendency to focus attention on negative stimuli. This can lead to more in-depth processing of negative information and therefore better recognition memory (Ortony et al., 1983). Similarly, the Negative Emotional Valence Enhances Recapitulation (NEVER) model (Bowen et al., 2018) further highlighted the role of negative valence in enhancing the reactivation of sensory details over time, suggesting that already in early ontogeny negative events and stimuli may be encoded and retrieved with greater sensory fidelity (Vaish et al., 2008). Although both positive and negative advantages have been reported, it is difficult to draw clear conclusions regarding directionality, not least because different studies use different methods and might therefore tap into different processes.

Although individual word valence has been shown to influence word learning and processing, understanding how people acquire the valence of unfamiliar words requires further investigation. One possible mechanism is through the emotional tone of the context in which the word appears. Word valence is usually determined from large-scale rating studies where participants rate the valence of individual words on a scale (e.g., Warriner et al., 2013). Affective ratings can be predicted from contextual variables such as contextual diversity (Recchia & Louwerse, 2015), and Kuhlmann et al. (2017) found that the valence of semantic neighbours within a word’s associative network influences the perceived valence of neutral words. This led them to consider valence as a “semantic super-feature.” In line with this, Snefjella and Kuperman (2016) reported a positive correlation between word valence and contextual valence. This suggests that words acquire a valence that reflects the overall emotional tone of the contexts in which they are used. They also found that the contextual valence of a word predicts lexical decision performance, even when the influence of word valence was controlled, reinforcing the idea that a word’s contextual history influences lexical processing (Hsiao et al., 2020).

From this background, experiments have investigated the influence of emotional context on the learning of new words. Snefjella et al. (2020) asked native speakers of English to learn nine novel nouns (e.g., *plurk* as a substitute for “the real word”), each embedded in five short passages that were designed to be neutral, negative, or positive. In the subsequent post-tests, participants showed clear evidence of word form and meaning learning, with the positive condition resulting in the best learning outcomes. Importantly, all the novel words acquired emotional connotations, suggesting that there was a transfer of valence from contexts. Using the same paradigm, Lana and Kuperman (2024) investigated the learning of novel words that denoted either abstract (e.g., religion) or concrete nouns (e.g., tool). In line with Snefjella et al. (2020), they found that positive contexts boosted word learning overall, but a contextual transfer of valence was only evident for concrete, but not abstract nouns. Although other experiments have investigated word learning in varying emotional contexts, findings are difficult to compare as different methods have been used. For example, Driver (2022) found better word learning when novel words denoted neutral concrete meanings and when novel words were embedded in neutral or negative emotion-laden texts, yet Frances et al. (2020) reported a facilitative effect for novel concrete words learned in positive contexts in relation to neutral contexts; note however they did not include any negative contexts. Taken together, these studies consistently show that a novel concrete word can acquire valence from the emotional tone of its surrounding text. Less clear, however, is whether this generalises to abstract words and whether positive or negative contexts (or both) support word learning, and why.

Existing studies have focused predominantly on nouns. Plausibly, words from other grammatical classes, such as adjectives, might show a different pattern. Compared with nouns, adjectives tend to be more abstract, and their meanings might be more context-dependent than nouns (Davies et al., 2020; Dawson et al., 2021). From a language evolution perspective, adjectives are more robustly associated with valence-dependent mutation compared with nouns (Jackson et al., 2023). It is thus likely that the effect of contextual valence may be more important for this word class. We therefore focused on novel adjectives and used a naturalistic reading procedure to investigate the effect of contextual valence on word learning. We asked whether people learn the valence of novel adjectives from positive, neutral, and negative contexts immediately after reading. We also asked how well people remembered the newly learned words 24 hr later. This allowed us to investigate three hypotheses across the two test points:

Hypothesis 1: Participants would learn novel word forms from reading short narratives, especially in more emotional (positive and negative) contexts.
Hypothesis 2: Participants would infer novel word meanings from reading short narratives, especially in more emotional (positive and negative) contexts.
Hypothesis 3: Participants would infer the valence of novel words from the linguistic context in which they appear.

## Method

### Participants

Eighty-seven participants (42 females, 45 males) were recruited through Prolific and completed both sessions remotely. An additional seven participants completed Session 1 only and did not return for Session 2, hence their data were omitted from any analyses. The ages of the 87 participants who completed both sessions of the study ranged from 18 to 30 years (*M*_age_ = 25.72, *SD*_age_ = 3.27). All participants reported to be native English speakers based in the United Kingdom have normal or corrected-to-normal vision, and no history of dyslexia or other language difficulties. They all provided consent before taking part. Following our pre-registered exclusion criterion, 11 participants were excluded from all the analyses due to them failing more than 20% of the attention cheques. The final sample size was 76 (34 females, 42 males; range 19–30 years, *M*_age_ = 25.58, *SD*_age_ = 3.30). They received a £7.5 payment for their participation via Prolific.

### Design

There was one independent variable, contextual valence, with three levels: neutral, negative, and positive. This was manipulated among participants. Accuracy and reaction time (RT) were measured and served as dependent variables. The study spanned two sessions. Session 1 consisted of a reading phase and an immediate test phase. Session 2 consisted of a delayed test phase only and was available 24 hr after participants completed Session 1. The study, including the sample size, exclusion criteria, and confirmatory analysis plan, was pre-registered ahead of data collection (https://osf.io/sc4ze/).

### Materials

We created 60 naturalistic narratives (*M*_word count_ = 18.95, *SD*_word count_ = 2.65) of either positive, neutral, or negative valence (20 narratives in each condition). The sentiment of each narrative was estimated using a *Bidirectional Encoder Representations from Transformers (BERT)* model, a transformer-based machine learning technique for natural language processing (Devlin et al., 2019). *BERT* considers the entire context of words in a sentence, rather than one word at a time, allowing it to capture emotional nuances in the narratives (see the Online Supplementary Materials for more details of the approach). On a scale of −1 (very negative) to 1 (very positive), the mean estimated sentiment was −0.41 (*SD* = 0.43) for the narratives in the negative context, −0.12 (*SD* = 0.40) in the neutral context, and 0.77 (*SD* = 0.13) in the positive context. These narratives were also rated by a group of 19 native English speakers, who did not participate in the main study, on a Likert-type scale of 1 to 7, how positive/negative each narrative made them feel. Negative narratives had a valence rating of 1.95 (*SD* = 0.57), followed by 4.11 (*SD* = 0.18) for the neutral, and 5.92 (*SD* = 0.61) for the positive narratives. Pearson correlation shows that the valence ratings by the 19 participants correlated strongly and positively with the *BERT* estimated sentiments, *r* = 0.69, *p* < .001. Both approaches showed that contextual valence differed significantly across the three valence conditions, with narratives in the positive condition showing the most positive valence, followed by neutral, and then by negative. In addition, another group of 20 native English speakers further categorised whether they thought the narratives were neutral, negative, or positive. The mean agreement rate between participants’ categorisation and the predetermined categorisation was 96.75% (*SD* = 17.7%). No item had an agreement rate below 80%. Narratives across conditions were matched for their overall word count (*M* = 18.95, *SD* = 2.68) and mean length of utterance (*M* = 8.72, *SD* = 1.97). A sample set of narratives is shown in Table 1 (see Supplementary Materials for all narratives and computations).

**Table 1.** Example narratives in each contextual valence condition.

Contextual valence	Examples
Neutral	This sopable machine was newly produced by the company. It has a sopable cover and four wheels.
Negative	I had a plarous argument with my friend today. We could not agree. Their plarous words hurt my feelings.
Positive	I am having a picial time with my family in this beautiful weather. We enjoyed the picial scenery.

There were 70 novel words, 30 of which were target novel words and the others were foils. Each of the 30 target novel words was read by participants in two narratives of the same valence. Within each narrative, the target word appeared twice (see Table 1). The novel words were six or seven letters long, *M* = 6.63 letters, *SD* = 0.46. They do not have a base meaning and were created to have a nonword stem plus an adjective suffix, for example, the nonword stem “rar” and the adjective suffix “ive” led to the novel word “rarive.” We chose 10 adjective suffixes from a list of suffixes with high diagnosticity values for adjectives, as calculated by Ulicheva et al. (2020). The target novel words had no replacement orthographic neighbours, according to *NWatch* (Davis, 2005). Assignment of target novel words to the contextual valence condition was counterbalanced such that a novel word that appeared in the positive context for one participant appeared in the neutral or negative context for other participants. The foils were the same across participants.

### Procedure

**Figure 1.**
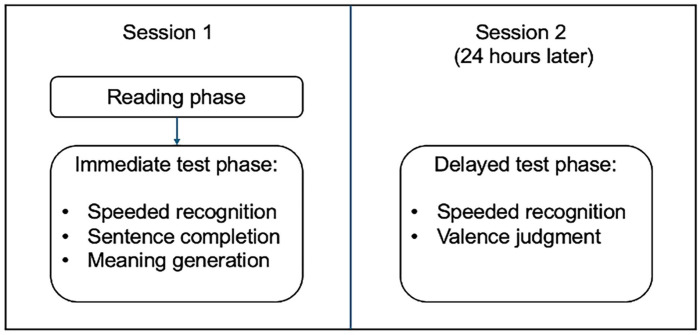
Procedure of the two-session study.

Participants signed up for the two-session study on Prolific. After reviewing the participant information sheet and providing consent, participants reported basic demographic data. The experiment was programmed and hosted on Gorilla Experiment Builder (www.gorilla.sc, Anwyl-Irvine et al., 2020). The procedure of the experiment is shown in Figure 1.

(i) Reading phase. This was structured around the premise of an alien attempting to learn English who occasionally replaced English words with words from its own language when writing a diary. These served as the novel words for the purposes of the word-learning experiment. Participants saw the novel words in neutral, negative, or positive narratives written, and each participant experienced 30 novel words embedded in 60 short narratives, evenly split into two blocks of 30. They were told to pay attention to these novel words. Each block also contained five narratives that served as attention cheques, where participants answered a comprehension question (in a multiple-choice format) based on the narrative they just read. Participants who failed 20% of these cheques (*N* = 11) were excluded from all analyses.
(ii) Immediate test phase. Immediately after reading the narratives, word learning was assessed via three outcome measures administered in a fixed order: speeded recognition, sentence completion, and meaning generation. For *speeded recognition*, modelled after lexical decision, participants identified whether they had previously seen a presented letter string. Each trial began with a fixation cross displayed for 250 ms, followed by the letter strings, during which the participants pressed buttons on the keyboard to make a judgement. The next trial began as soon as a response was recorded. Accuracy and RT were recorded. Each participant responded to 60 items in total (30 target novel words and 30 distractors), presented in a random order in a single block. This task assessed whether participants could learn the novel word form.

In *sentence completion*, each trial showed a sentence with a missing word, and participants were asked to select the best completion for the sentence from a choice of three novel words they had encountered in the reading phase (one from each valence condition). Thirty sentences of either neutral, negative, or positive valence were created for this task, 10 in each of the valence conditions and each missing one word. The sentences were different from the narratives in the reading phase, but they followed similar themes and valence as those narratives. Each trial began with a fixation cross displayed for 250 ms, followed by the sentence and the three options displayed below. Participants made a judgement by clicking on the most suitable novel word option. For example, if the sentence is of positive valence, the participants would select one of the novel words that previously appeared in a positive narrative context during the reading phase. The next trial began as soon as a response was recorded. Accuracy and RT were recorded, and no feedback was provided. The 30 trials were presented in a random order in a single block. This task assessed whether participants could learn the novel word meaning, assessed through valence.

The final task was *meaning generation*. In each trial, participants were shown one of the 30 newly learnt words (presented in a randomised order), and they were required to type in an English word they considered to correspond with the meaning of the novel word. The words produced were cross-referenced with norms of valence for English lemmas (Warriner et al., 2013) and these values were used to assign a valence score to each response. The outcome variable is the valence of the produced words (ranging from one to nine, with one being very negative and nine being very positive), rather than a more fine-grained meaning. The task assessed whether participants could infer the word’s valence from context.

At the end of Session 1, participants completed a brief questionnaire soliciting their perceptions of the experiment, their reading strategies, and any additional comments (see Supplementary Materials). They were reminded that there would be a delayed test available 24 hr later, but they were not told what tests would be administered. The reading phase and the immediate test phase in Session 1 took around 30 min to complete.

(iii) Delayed test phase. Twenty-four hours later, participants completed an identical version of *speeded recognition* to Session 1, with trials appearing in random order. They then completed a *valence judgement* task (which was not administered in Session 1) in which they made a categorical judgement to indicate whether a novel word (experienced in the reading phase) was positive, neutral, or negative. This task mentioned the concept of valence explicitly; hence it was only administered at the end, so it would not influence participants’ performance on other tasks. Each word was displayed for 300 ms before the three options appeared. Participants made a judgement by clicking on the most suitable option. The next trial started as soon as a response was recorded. Accuracy and RT were recorded. There were 30 trials in total, presented in a random order in a single block. The delayed test phase in Session 2 required 10 min to complete. This task assessed whether participants could learn the novel word meaning, assessed through valence.

### Statistical analyses

As pre-registered, we fitted mixed-effect models with random effects for participants and stimuli, using the “lme4” package (Bates et al., 2015) in R (R Core Team, 2022). RT data for the correct trials were transformed to give a more normal residual distribution, based on the suggestion of the Box–Cox procedure (Box & Cox, 1964) and inspection of the qqplot (Millard, 2013). For all full models DV ~ ContextualValence + (1 + ContextualValence—participant) + (1 + ContextualValence—item) that failed to converge, we followed Mak, Curtis, et al. (2023) and Mak, O’Hagan, et al. (2023) by simplifying the random-effect structure using the R package “buildmer” (Voeten, 2023). Unless otherwise specified, after simplification, a binary logistic mixed-effects model was adopted for the dependent variable accuracy (1 or 0) and included the fixed effect of contextual valence and by-participant and by-stimuli random intercepts. A linear mixed model was fitted to the transformed RTs, with contextual valence as the sole fixed effect, and random intercepts for participants and stimuli. A likelihood ratio test was used to compare the full model to the reduced model to assess whether including the fixed factor ContextualValence significantly improved model fit.

The fixed effect was contextual valence (neutral, negative, or positive). This was dummy-coded, and the neutral condition served as the reference level, yielding two comparisons: neutral vs. positive and neutral vs. negative. Models were fitted using maximum likelihood estimates. Following these confirmatory analyses, we also conducted an exploratory analysis comprising a direct comparison between the positive and negative conditions. Data and analysis scripts are accessible via Open Science Framework (OSF: https://osf.io/r6hx9/).

## Results

Mean performance in each post-test is summarised in Table 2.

**Table 2.** Descriptive statistics (*M* and *SD*) of performance in immediate and delayed post-tests.

Task	Speeded recognition (immediate)		Sentence completion (immediate)		Meaning generation (immediate)	Speeded recognition (delayed)		Valence judgement (delayed)	
Measured variable	Accuracy	RT	Accuracy	RT	Valence score	Accuracy	RT	Accuracy	RT
Neutral	0.71 (0.18)	853 (276)	0.49 (0.18)	5,078 (3,027)	5.62 (0.63)	0.78 (0.15)	831 (341)	0.44 (0.20)	1,409 (1,019)
Negative	0.79 (0.15)	835 (203)	0.53 (0.17)	4,207 (1,710)	4.89 (0.73)	0.82 (0.15)	826 (315)	0.37 (0.17)	1,371 (719)
Positive	0.76 (0.17)	835 (230)	0.48 (0.21)	4,786 (2,924)	5.98 (0.69)	0.80 (0.16)	832 (346)	0.45 (0.17)	1,291 (970)

RT is measured in milliseconds (ms); Accuracy is a probability from 0 to 1; Valence score is a continuous value between 1 and 9 based on Warriner et al. (2013), with larger values indicating greater positivity, and smaller values indicating greater negativity. The chance level is 0.5 for speeded recognition, and 0.33 for sentence completion and valence judgement.

### Hypothesis 1: word learning assessed by speeded recognition

Word form learning was assessed via *speeded recognition* in both sessions. Starting with the immediate post-test data, we first computed sensitivity from raw responses to the speeded recognition task (number of hits, false alarms, correct rejection, and misses) using the *dprime* function in the “psycho” R package (Makowski, 2018). A one-sample *t*-test provided clear evidence that participants could distinguish learned items from distractors, *M_d′_* = 1.49, 95% confidence interval (CI) = [1.35, 1.63], *t*(75) = 21.30, *p* < .001.

**Figure 2.**
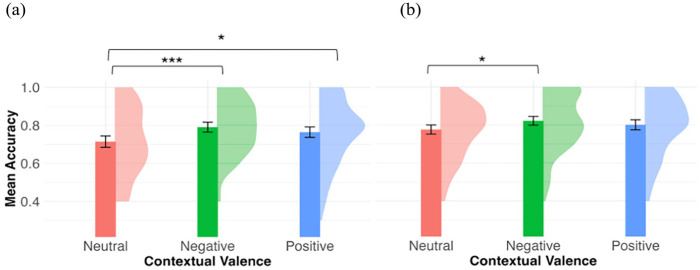
Mean recognition accuracy by contextual valence in immediate post-test (a) and delayed post-test (b). The density plots represent the distribution of the mean accuracy. Error bars represent 95% within-participant confidence intervals. ***p < .001, **p < .01, *p < .05.

We then compared novel words learned across the different valence conditions. Figure 2a shows the mean recognition accuracy by contextual valence in the immediate post-test. From the likelihood ratio test, contextual valence was a significant predictor for recognition accuracy, χ^2^(2) = 12.57, *p* = .002. As compared with words in the neutral context (*M* = 0.71, *SD* = 0.18), participants were more accurate in recognising words experienced in the negative context (*M* = 0.79, *SD* = 0.15) and the positive context (*M* = 0.76, *SD* = 0.17; negative vs. neutral: β = 0.44, *SE* = 0.13, *z* = 3.50, *p* < .001; positive vs. neutral: β = 0.28, *SE* = 0.12, *z* = 2.26, *p* = .02).

We also tested for the differences in RT in the three valence conditions at immediate *speeded recognition*. Of all “hit” trials (*N* = 1,725), 24 trials (1.3%) with RTs that were > 3 SDs away from the mean RT of that participant were removed. The remaining RT data were inversely transformed to give a more normal residual distribution. There was no significant effect of contextual valence on RT, χ^2^(2) = 0.87, *p* = .65. Compared with words learned in the neutral context (*M* = 853 ms, *SD* = 276), there were no significant differences between novel words learned in the negative context (*M* = 835 ms, *SD* = 203) or the positive context (*M* = 835 ms, *SD* = 230; negative vs. neutral: β = −0.013, *SE* = 0.016, *t* = 0.80, *p* = .42; positive vs. neutral: β = −0.013, *SE* = 0.016, *t* = 0.82, *p* = .41).

Mirroring the findings from the immediate recognition test, there was evidence that participants could distinguish learned items from distractors in the delayed recognition test 24 hr after learning, *M_d′_* = 1.63, 95% CI = [1.50, 1.75], *t*(75) = 25.5, *p* < .001. Accuracy data are shown in Figure 2b. The fixed factor contextual valence was marginally significant, χ^2^(2) = 5.81, *p* = .05. Compared with words in the neutral context (*M* = 0.78, *SD* = 0.15), participants were more accurate in recognising words experienced in the negative context (*M* = 0.82, *SD* = 0.15), β = 0.33, *SE* = 0.14, *z* = 2.43, *p* = .02. There was no difference between the positive (*M* = 0.80, *SD* = 0.16) and neutral condition (β = 0.18, *SE* = 0.13, *z* = 1.37, *p* = .17).

Of all hit trials (*N* = 1,824), 39 (2.1%) had an RT > 3 SDs away from the participant’s mean RT and were hence removed. The fixed effect of contextual valence was not significant, χ^2^(2) = 0.86, *p* = .65. Compared with words appearing in neutral valence paragraphs (*M* = 831 ms, *SD* = 341), there were no significant differences between RT towards novel words in the negative context (*M* = 826 ms, *SD* = 315) or the positive context (*M* = 832 ms, *SD* = 346; negative vs. neutral: β = −0.015, *SE* = 0.016, *t* = 0.92, *p* = .36; positive vs. neutral: β = −0.0053, *SE* = 0.016, *t* = 0.33, *p* = .74).

In summary, the results of *speeded recognition* supported Hypothesis 1 that participants can learn novel word forms from reading short narratives, as indexed by the above-chance sensitivity in distinguishing learned items from distractors in both sessions. Participants were more accurate in emotional (positive and negative) contexts in the immediate post-test, and more accurate only in the negative context in the delayed post-test. There were no significant contextual valence effects in RTs.

### Hypothesis 2: word meaning learning assessed by sentence completion and valence judgement

Word meaning learning was assessed with *sentence completion* in the immediate post-test and *valence judgement* in the delayed post-test. To test learning of novel word meanings in *sentence completion*, we first compared a participant’s response accuracy with chance performance, which was 0.33. Using a one-sample *t*-test, there was clear evidence that participants’ performance was above chance, *M*_accuracy_ = 0.50, 95% CI = [0.47, 0.53], *t*(75) = 9.86, *p* < .001. The likelihood ratio test shows that contextual valence was not a significant predictor of sentence completion accuracy, χ^2^(2) = 1.24, *p* = .54. As compared with the neutral context (*M* = 0.49, *SD* = 0.18), there was no difference in accuracy for the negative context (*M* = 0.53, *SD* = 0.17), or the positive context (*M* = 0.48, *SD* = 0.21; negative vs. neutral: β = 0.14, *SE* = 0.17, *z* = 0.84, *p* = .40; positive vs. neutral: β = −0.04, *SE* = 0.17, *z* = −0.24, *p* = .81).

Of all the correct trials (*N* = 1,140), those more than 3 SDs from the mean RT for that participant were removed (*N* = 9, 0.8%). The likelihood ratio test shows that the fixed effect of valence was a significant predictor of log-transformed sentence completion time, χ^2^(2) = 7.19, *p* = .03. Compared with RTs for neutral sentences (*M* = 5,078 ms, *SD* = 3,027), participants responded faster in negative (*M* = 4,207 ms, *SD* = 1,710), but not positive contexts (*M* = 4,786 ms, *SD* = 2,924; negative vs. neutral: β = −0.13, *SE* = 0.04, *t* = −2.88, *p* = .004; positive vs. neutral: β = −0.07, *SE* = 0.04, *t* = −1.52, *p* = .13).

**Figure 3.**
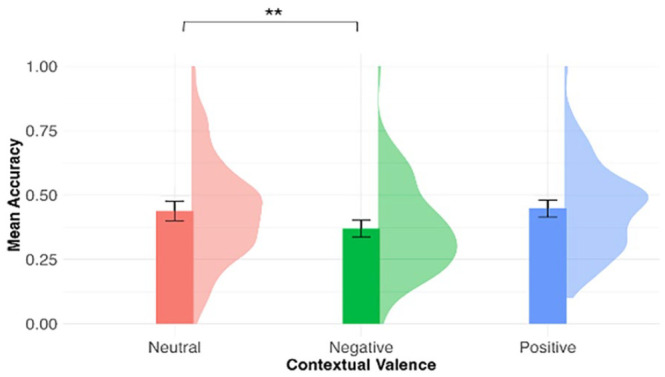
Mean valence judgement accuracy by contextual valence in the delayed post-test. The density plots represent the distribution of the mean accuracy. Error bars represent 95% within-participant confidence intervals. ***p < .001, **p < .01, *p < .05.

As above, a one-sample *t*-test showed that participants’ performance was above chance (0.33) in *valence judgement* in the delayed post-test, *M*_accuracy_ = 0.42, 95% CI = [0.39, 0.45], *t*(75) = 6.40, *p* < .001. Figure 3 shows the mean valence judgement accuracy by contextual valence. Contextual valence was a significant predictor of valence judgement accuracy, χ^2^(2) = 11.70, *p* = .003. As compared with novel words learned in the neutral contexts (*M* = 0.44, *SD* = 0.20), novel words in the negative conditions were less accurately judged as negative (*M* = 0.37, *SD* = 0.17), β = −0.30, *SE* = 0.11, *z* = −2.77, *p* = .006. There was no significant difference between the neutral and positive conditions (*M* = 0.45, *SD* = 0.17), β = 0.04, *SE* = 0.11, *z* = 0.36, *p* = .72.

Of all 954 correct trials, those more than 3 SDs away from the mean RT for that participant were removed (*N* = 8, 0.8%). A linear mixed-effects model was used with inverse square root-transformed RT as the dependent variable and included the fixed effect of contextual valence and random intercepts and slope for participants. Contextual valence was a significant predictor for RT, χ^2^(2) = 10.02, *p* = .007. Yet, compared with words experienced in the neutral context (*M* = 1,409 ms, *SD* = 1,019), there was no difference in RT for words learned in the negative context (*M* = 1,371 ms, *SD* = 719), or positive contexts (*M* = 1,291 ms, *SD* = 970; negative vs. neutral: β = 0.69, *SE* = 0.68, *t* = 1.03, *p* = .30; positive vs. neutral: β = −1.11, *SE* = 0.68, *t* = −1.62, *p* = .10).

These results partially support Hypothesis 2, that participants can infer novel word meanings from reading short narratives. Performance was above chance in both *sentence completion* and *valence judgement*. However, participants were not consistently more accurate or faster in the emotional contexts. We return to discuss this finding later.

### Hypothesis 3: word valence inference assessed by meaning generation

**Figure 4.**
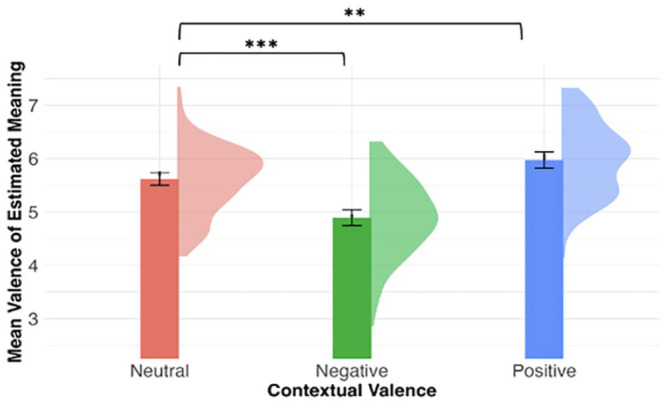
Mean valence scores of generated meanings (immediate post-test) per participant by contextual valence. The density plots represent the distribution of the mean estimated valence. Error bars represent 95% within-participant confidence intervals. ***p < .001, **p < .01, *p < .05.

Most responses (1,934 out of 2,280; 84.8%) had valence scores listed in Warriner et al.’s (2013) norms. Reasons for the absence of associated valence included random letter strings, “?”, more than one word, or the response word was not normed. Figure 4 shows the mean valence score of generated meaning per participant by contextual valence. We built a linear mixed model with the valence score as the dependent variable and included the fixed effect of contextual valence and a by-item random slope and random intercepts. The likelihood ratio test shows that contextual valence was a significant predictor of estimated valence scores, χ^2^(2) = 33.99, *p* < .001. Compared with novel words appearing in the neutral context (*M* = 5.62, *SD* = 0.63), participants assigned more negative meanings to novel words experienced in the negative context (*M* = 4.89, *SD* = 0.73), and more positive meanings to novel words experienced in the positive context (*M* = 5.98, *SD* = 0.69; negative vs. neutral: β = −0.72, *SE* = 0.15, *t* = −4.66, *p* < .001; positive vs. neutral: β = 0.36, *SE* = 0.12, *t* = 2.98, *p* = .003).

### Interim summary of confirmatory analyses

Hypothesis 1 that participants can learn novel word forms from reading short narratives was supported by the results of *speeded recognition* in both immediate and delayed post-tests. Participants were more accurate in emotional (positive and negative) contexts in the immediate post-test in Session 1, and more accurate only in the negative context in the delayed post-test in Session 2. Hypothesis 2 that participants can infer novel word meanings from reading short narratives was partially supported by the above-chance performance of *sentence completion* and *valence judgement*. Compared with neutral conditions, there was no difference in positive conditions in the two tasks. Participants were faster in accurately completing negative sentences, but they were less accurate when judging novel words to be negative in valence judgement. Hypothesis 3 about word valence inference was supported by the results of *meaning generation*, where the valence of generated meanings reflected the relative emotional valence of the context in which the novel words appeared.

The inconsistent results of *sentence completion* in the immediate post-test and *valence judgement* in the delayed post-test regarding the responses to negative options prompted us to conduct a post hoc analysis to explore whether participants exhibited a preference for options of a specific valence, regardless of accuracy. Rather than calculating the accuracy of responses for each condition and only considering RTs for correct trials, we combined both correct and incorrect trials to look at the proportion of times an option of a particular valence was selected.

Furthermore, while we were mainly interested in the influence of emotional versus neutral context and did not hypothesise for the directionality of the influence of valence, we conducted a post hoc comparison between positive and negative contexts.

### Exploratory analysis

To explore the directionality of the influence of valence, we conducted a post hoc comparison between positive and negative contexts. We found that the difference between the two was significant only in *meaning generation* and *valence judgement*. In *meaning generation*, as compared with novel words appearing in the negative context, participants assigned a more positive meaning to novel words experienced in the positive context (β = 1.08, *SE* = 0.14, *t* = 7.73, *p* < .001). In *valence judgement*, positive words were judged faster and more accurately than negative words (accuracy: β = 0.33, *SE* = 0.11, *z* = 3.13, *p* = .002; RT: β = −1.68, *SE* = 0.56, *t* = 3.01, *p* = .003). The difference in accuracy and RT between positive and negative conditions in *speeded recognition* and *sentence completion* did not reach significance (*p*s > .05).

We then conducted a post hoc analysis of possible option selection preference, to better understand the inconsistent results of *sentence completion* in the immediate post-test and *valence judgement* in the delayed post-test regarding the responses to negative options. It allowed us to explore whether participants might exhibit a preference for options of a specific valence in these three alternative forced-choice tasks, regardless of accuracy. In contrast to the confirmatory analysis, the dependent variable was option selection proportion, which was the proportion of times out of all trials, a negative, neutral, or positive option was selected. The option selection proportion summed up to 1 across all three valence conditions. A linear model was adopted with option selection proportion as the dependent variable and included the predictor of option valence, which had three levels, negative, neutral, and positive. In *sentence completion*, option valence was not a significant predictor for the proportion of times an option was selected, *F*(2, 225) = 0.07, *p* = .93. As compared with neutral options (novel words that were encountered in neutral contexts; *M* = 0.34, *SD* = 0.08), there was no difference in preference for the negative options (*M* = 0.33, *SD* = 0.07) or the positive options (*M* = 0.33, *SD* = 0.07; negative vs. neutral: β = −0.0009, *SE* = 0.01, *t* = −0.07, *p* = .94; positive vs. neutral: β = −0.004, *SE* = 0.01, *t* = −0.36, *p* = .72). For the analysis of RT, option valence was a significant predictor of log-transformed RT, χ^2^(2) = 10.22, *p* = .007. As compared with the novel words previously encountered in the neutral context (*M* = 4,898 ms, *SD* = 2,230), novel words previously experienced in the negative context (*M* = 4,501 ms, *SD* = 1,881) were selected faster, β = −0.06, *SE* = 0.02, *t* = −3.14, *p* = .002. Difference between positive (*M* = 4,838 ms, *SD* = 2,483) and neutral options were not significant, β = −0.02, *SE* = 0.02, *t* = −0.90, *p* = .37.

**Figure 5.**
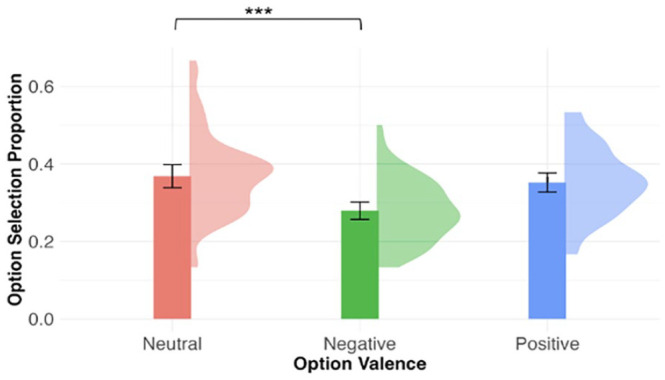
Option selection proportion by option valence in valence judgement (exploratory analysis). The density plots represent the distribution of the mean option selection proportion. Error bars represent 95% within-participant confidence intervals. ***p < .001, **p < .01, *p < .05.

In *valence judgement*, where participants indicated explicitly whether the novel word presented was neutral, negative, or positive, option valence was a significant predictor of the option selection proportion, *F*(2, 225) = 20.16, *p* < .001. Figure 5 shows the mean option selection proportion by option valence. The *y*-axis shows the proportion of times out of all trials when a negative, neutral, or positive option was selected. The option selection proportion for the three valence conditions, represented in the heights of the three bars, summed up to 1. As compared with neutral options (*M* = 0.37, *SD* = 0.11), negative options (*M* = 0.28, *SD* = 0.08) were selected less of the time, but the difference between positive (*M* = 0.35, *SD* = 0.09) and neutral options were not significant (negative vs. neutral: β = −0.09, *SE* = 0.01, *t* = −5.96, *p* < .001; positive vs. neutral: β = −0.02, *SE* = 0.01, *t* = −1.09, *p* = .28). For the analysis of RT, linear mixed model was used with inverse square root-transformed RT as the dependent variable and included the fixed effect of option valence and random intercepts for participants and stimuli. Option valence was a significant predictor of reaction times, χ^2^(2) = 8.35, *p* = .02. As compared with reaction times to the neutral options (*M* = 1,359 ms, *SD* = 889), negative options (*M* = 1,376 ms, *SD* = 722) were selected significantly slower, but the difference between positive (*M* = 1,329 ms, *SD* = 775) and neutral options were not significant (negative vs. neutral: β = 0.87, *SE* = 0.36, *t* = 2.44, *p* = .01; positive vs. neutral: β = −0.10, *SE* = 0.34, *t* = −0.29, *p* = .77).

## Discussion

Our study examined if and how emotional contexts, varying in valence, influence the learning of novel words, and whether word valence can be inferred from contextual valence. Participants learned novel words embedded in short narrative contexts of either positive, neutral, or negative valence. We found that across the different contexts, participants were able to distinguish target novel words from distractors, both in the immediate and delayed post-tests. As compared with words learned in the neutral context, participants were more accurate in recognising word forms learned in the negative context (in both immediate and delayed post-tests), and the positive context (only in the immediate post-test). Participants were faster in negative sentence completion, but less accurate when making negative valence judgements. We also found that people inferred the valence of novel words from the linguistic context in which they appeared, where the valence of generated meanings reflected the relative emotional valence of the context in which the novel words appeared.

Our findings that participants estimated the valence of novel words after brief exposure aligned with existing research. Following the general correlation between contextual valence and word valence across a large corpus of text (Snefjella & Kuperman, 2016) and the suggestion that the valence of the word might be inferred from its context, Snefjella et al. (2020) and Lana and Kuperman (2024) provided initial experimental evidence for the transfer of valence to novel concrete nouns. Our study supports and extends this finding to adjectives, which tend to be more abstract and emotionally charged. Furthermore, while both Snefjella et al. (2020) and Lana and Kuperman (2024) used a valence rating task, our study employed a novel task that did not probe valence directly. Instead, we asked people to capture the meaning of each novel word in one word. We then obtained the valence of the response words from existing norms. This provided evidence for the implicit transfer of contextual valence to novel adjectives, without having to ask participants to explicitly reflect on valence directly. A different type of interpretation is that participants actively associated the novel word forms with existing words during the reading phase of the study. This might be thought of as akin to second language learning, whereby the difference in valence attributed to a novel word reflects the already known word to which the novel word is associated. While plausible, this does not explain our results entirely. For one, the novel adjectives did not have a predefined meaning that could be simply translated or mapped to a familiar word. Note also that in the meaning generation task, participants were not provided with the narrative contexts. Instead, they had to reflect on the context of each newly learned novel word, and then find an English word that captures this understanding of meaning in context. We suggest, therefore, that the contextual valence experienced during the initial reading lingered and influenced their performance on the post-tests, which is reflected in the meaning generation task. This successful inference of valence from contexts to novel words supports the affective embodiment account, which suggests that emotional content aids in grounding abstract word meanings in affect, allowing for the development of lexical representations from linguistic rather than physical experiences (Vigliocco et al., 2014). This has implications for language acquisition for abstract words, which often rely on emotional cues for meaning since they lack direct sensorimotor connections (e.g., Borghi et al., 2017; Kousta et al., 2011; Ponari et al., 2018).

Our results suggest that both word form and their meanings can be learned from reading short narratives. For word learning, participants showed above-chance sensitivity to distinguish learned items from distractors in both immediate and delayed *speeded recognition*. This corroborates previous research showing that even a few encounters with a novel word during narrative reading may result in robust learning of visual word forms (e.g., Hulme et al., 2023; Mak et al., 2021). We found that participants were more accurate (but not faster) in recognising words learned in emotional contexts (both negative and positive) in the immediate post-test. Turning to word meaning, participants showed above-chance accuracy in both *sentence completion* and *valence judgement*. In *sentence completion*, where participants selected the best completion for the sentence from a choice of three novel words they had encountered in the reading phase, there was no difference in accuracy between different valence conditions. However, contextual valence was a significant predictor for reaction times for correct trials, and negative items were responded to faster. Note that while sentence completion was set up to be a task that tapped into meaning learning, we recognised that it assessed valence-based meaning.^1^ We are therefore not sure whether the participants were learning through meaning or valence, or a mix of both. We suggest that future studies could have a test of meaning that is not associated with valence. Together, these results provide further evidence for the affective embodiment account for word meaning acquisition and the role of emotional valence in providing an embodied learning experience in which to anchor abstract meanings (Ponari et al., 2018, 2020; Snefjella et al., 2020). From this view, the emotional content of the context aids in grounding abstract word meanings in emotional experiences. Our study focused specifically on adjectives, which tend to be more abstract than nouns and therefore perhaps more dependent on emotional cues that offer a grounding that would otherwise be unavailable from sensorimotor experiences.

Although not part of our initial hypotheses, some of our findings point to a negativity advantage. In *speeded recognition*, we found that after a 24 hr delay, words learned in the negative context were recognised more accurately compared with words learned in the neutral context. Similar results were found in *sentence completion*, where participants saw a sentence with a missing word and selected the best completion word option from three novel words that they had encountered in the reading phase (one from each valence condition). We found that as compared with new neutral sentences, participants were faster to complete new negative sentences with the correct word option that was initially encountered in a negative context in the reading phase. The word option that carries a negative connotation might be more attention-grabbing and faster to respond to. These negative completion word options might also make other options slower to respond to in non-negative sentence contexts. This finding suggests a subtle yet significant processing advantage for negatively valenced information (Vaish et al., 2008). Thus, the sustained negativity advantage in delayed *speeded recognition* and the faster processing of negative words in *sentence completion* suggests that negative information is recognised and remembered better (Ortony et al., 1983). The NEVER model (Bowen et al., 2018) highlights the role of negative valence in enhancing the reactivation of sensory details, consistent with negative events being encoded and retrieved with greater sensory fidelity (Vaish et al., 2008). This is in contrast to the positivity advantage suggested by the information density hypothesis (Unkelbach et al., 2008). This might be because a one-day delay in our study and that participants only experienced these novel words in two narratives might not be sufficient for novel words in the positive condition to become integrated into the existing lexicon. In comparison, Snefjella et al. (2020) and Lana and Kuperman (2024) found a consistent positivity advantage for newly learned words, but this was after five exposure opportunities and across a one-week interval. The amount of exposure and its time course might explain differences across studies. Another possible explanation for the deviations from the previous positivity advantage found in Snefjella et al. (2020) and Lana and Kuperman (2024) might be due to the nature of the target words, which are adjectives in the current experiment. From a language evolution perspective, compared with nouns, adjectives are more robustly associated with valence-dependent mutation, suggesting that the meanings of negative adjectives are more differentiated because there are more of them, and they are acquired at a faster rate (Jackson et al., 2023). We might therefore be more adapted to learn novel adjectives that carry a negative connotation as compared with nouns. Moreover, adjectives tend to have more extreme emotional valence and have meanings that are generally more context-dependent than nouns (Davies et al., 2020; Dawson et al., 2021).

Against this, however, findings from our *valence judgement* task appear to contradict the negativity advantage discussed above. Participants were less accurate in categorising novel words as negative when they had previously appeared in a negative context. To reconcile the discrepancy, we propose that the divergence may stem from the explicit nature of the demands introduced by the valence judgement task. Although both *sentence completion* and *valence judgement* employed a three alternative forced choice format, with options representing each of the valence conditions, *valence judgement* explicitly asked participants to decide on a valence category (positive, neutral, or negative). Participants might thus engage in different cognitive processes compared with tasks where valence assessment was not explicit and was instead disguised under the probing of meaning. The difference in task demand was an active decision on our part, as was the ordering of the tasks: as *valence judgement* was the last task administered, the explicit instruction to think about emotional valence could not bias behaviour in earlier parts of the study. For this more explicit assessment of valence knowledge, accuracy was lowest for words previously encountered in negative contexts. Exploratory analysis of the valence category choices made by participants in the *valence judgement* further demonstrated that regardless of response accuracy, the negative category was least selected, and even when selected, responses were slow. This non-negative preference when an explicit valence judgement is required might indirectly support the Pollyanna hypothesis, which describes a universal tendency to use positive language more often (Boucher & Osgood, 1969; Dodds et al., 2015). From this perspective, when participants have to make a valence decision, they are more likely to judge words as positive than negative.

The mixed results in the literature regarding positivity and negativity advantages might partially stem from differences in task demands and experimental paradigms. Tasks vary in complexity and may tap into different processing mechanisms, leading to different observation of results (e.g., Hsiao et al., 2020; Mak et al., 2021). In our study, for example, *speeded recognition* is relatively straightforward, but *valence judgement* performance is tricky to interpret as participants might have different understandings of valence and adopt different benchmarks to judge a word as positive, neutral, or negative. In addition, the terms “positivity advantage” and “negativity bias” are often loosely employed in the literature and might manifest differently depending on the specific cognitive task. As Unkelbach et al. (2020, p. 119) noted: “It is not *a priori* clear what constitutes an advantage or disadvantage in a given situation.” In lexical decision, for example, positive words are processed faster, suggesting a positivity advantage. However, when semantic decision is concerned, negative stimuli may attract more attention and be processed more slowly, leading to longer processing times which supports an increased vigilance for negative stimuli, indicative of a negativity bias (e.g., Fiske, 1980; Gao et al., 2022; Vaish et al., 2008). Although there is an emotionality effect, valence effects might be at least partly task-specific (Crossfield & Damian, 2021). A systematic review exploring these phenomena across various task demands and paradigms would be useful, and future research should aim for precision in defining and contextualising these terms within specific experimental settings.

Overall, our study builds on existing research to investigate the influence of emotional narrative contexts on word learning and extends it to learning adjectives via naturalistic reading. Participants showed evidence of learning after just two exposures, especially for words learned in emotional contexts. Newly learned words also inherited valence from the linguistic context in which they appeared, which holds implications for how emotional knowledge builds during language acquisition. With this in mind, it is worth noting that research on the influence of emotional context in word learning is still in its infancy and mainly targets adults. One related developmental study by Ponari et al. (2020) examined similar themes of learning emotional valence in children, but they operationalised *context* differently and used explicit teaching. We know of no children’s study that mirrors the independent reading paradigm in adults, and that investigates how children learn the emotional properties of new words during natural reading. This represents a knowledge gap, given experience with written language provides opportunities to learn new words and abstract concepts (Nation et al., 2022), and that the valence of existing words can influence children’s word learning (e.g., Ponari et al., 2018). Although there is evidence for a positivity advantage in younger children, there is also evidence to suggest that the effect dissipates with age (Bahn et al., 2017; Ponari et al., 2018) and that older children write stories which are less positive (Dong et al., 2024). Hence, it would be interesting to extend our experimental approach to children.

## Supplemental Material

sj-docx-1-qjp-10.1177_17470218241308221 – Supplemental material for Learning new words via reading: The influence of emotional narrative context on learning novel adjectives Supplemental material, sj-docx-1-qjp-10.1177_17470218241308221 for Learning new words via reading: The influence of emotional narrative context on learning novel adjectives by Yuzhen Dong, Matthew HC Mak, Robert Hepach and Kate Nation in Quarterly Journal of Experimental Psychology

## Notes

1. We thank an anonymous reviewer for raising this.
