# Supplementary material for: Learning new words via reading: The influence of emotional narrative context on learning novel adjectives
Source: Q J Exp Psychol (Hove). 2024 Dec 26;78(10):2167–80. doi: 10.1177/17470218241308221 (PMC12432280; doi:10.1177/17470218241308221)
Supplement: sj-docx-1-qjp-10.1177_17470218241308221 – Supplemental material for Learning new words via reading: The influence of emotional narrative context on learning novel adjectives [file sj-docx-1-qjp-10.1177_17470218241308221.docx]

Supplementary Material for:

**Learning New Words via Reading: The Influence of Emotional Narrative Context on Learning Novel Adjectives**

**Yuzhen Dong ([yuzhen.dong@psy.ox.ac.uk](mailto:yuzhen.dong@psy.ox.ac.uk))**

Department of Experimental Psychology, University of Oxford, United Kingdom

**Matthew H. C. Mak ([matthew.mak@warwick.ac.uk](mailto:matthew.mak@warwick.ac.uk))**

Department of Psychology, University of Warwick, United Kingdom

**Robert Hepach ([robert.hepach@psy.ox.ac.uk](mailto:robert.hepach@psy.ox.ac.uk))**

Department of Experimental Psychology, University of Oxford, United Kingdom

**Kate Nation ([kate.nation@psy.ox.ac.uk](mailto:kate.nation@psy.ox.ac.uk))**

Department of Experimental Psychology, University of Oxford, United Kingdom

1. **All target novel words**

rarive

venvic

depish

kunable

raxous

jemical

quanful

binushy

supular

picial

higgive

roific

wapish

sopable

domous

paxible

priful

shromy

jafular

hitial

thutive

talulic

yeotish

bulable

plarous

vorical

spomful

phrinty

vatular

cretial

1. **Foils**

geosive

cusive

horive

shonbic

milatic

huxatic

thunish

tredish

tumish

gilable

gufable

hixable

merous

chutous

weerous

berical

temical

vebical

shurful

glaful

croiful

scemowy

chrabby

roithy

pimular

lonular

ferular

dracial

phutial

jetial

mibical

genful

mollive

weacial

hilish

vuxable

dolous

cedular

vepatic

celoppy

1. **Narrative Contexts**

| **Context Valence** | **Block** | **Blank Content** |
| --- | --- | --- |
| Positive | One | It was my birthday yesterday. I had a _____ cake together with some _____ friends. |
|  |  | My friends held a party in their _____ garden last week. It was a _____ day with warm sunshine and a gentle breeze. |
|  |  | I got myself a _____ cat! I love holding it in my arms. It is soft and _____. |
|  |  | It was _____ to see my best friends again after so many years. We had a _____ chat along the river. |
|  |  | I sat next to a _____ classmate today. She is really _____ and helped me a lot. |
|  |  | I won a _____ prize in writing today. I love the feeling of writing _____ stories. |
|  |  | This weekend, I had a _____ time at home. I watched a _____ movie and felt very relaxed. |
|  |  | I helped my grandma clean her house today. It feels _____ to be helpful. We had a _____ time together. |
|  |  | After working hard, I finally completed my homework. How _____. It feels _____ to be able to enjoy my life again. |
|  |  | I am having a _____ time with my family in this beautiful weather. We enjoyed the _____ scenery. |
| Neutral |  | I was reading the cookbook: "First wash the ingredients using _____ water and pour some oil into a _____ pan." |
|  |  | I went to the _____ library today. I first turned left at the _____ house. |
|  |  | I booked a flight ticket this morning. I clicked on the _____ icon on the website, and I selected the _____ button. |
|  |  | This _____ machine was newly produced by the company. It has a _____ cover and four wheels. |
|  |  | I drove my _____ way home today. Everything was normal, nothing happened. I am used to driving on this _____ road. |
|  |  | In the morning, I usually wake up on my _____ bed. I have cereal from my _____ bowl. |
|  |  | I am at the _____ train station and reading the timetable. The _____ train will leave at 7 PM. |
|  |  | I tried using this _____ kettle to boil water today. The _____ kettle was made of steel. |
|  |  | I wanted to return a book. I went to the _____ building and turned right when I reached the _____ door. |
|  |  | I went to see a _____ house today. It had several _____ rooms and a normal kitchen. |
| Negative |  | The _____ video games gave me a nightmare. The characters were fighting in a _____ way. |
|  |  | It is so _____ that the _____ teacher only scolded me today, when many others were also fighting. |
|  |  | I felt _____ when I saw the news about the _____ car accident. Several people were injured! |
|  |  | I failed again after many _____ attempts at the maths challenge. I felt so _____. |
|  |  | I had a _____ argument with my friend today. We could not agree. Their _____ words hurt my feelings. |
|  |  | I was _____ today after working nonstop for hours. I felt _____ when going through endless homework. |
|  |  | Someone said that I look _____, and they fear hanging out with a _____ monster like me. |
|  |  | I fell down the stairs and injured myself. How _____! My legs are _____ now. |
|  |  | This _____ place is giving me a headache. It is so _____ to hear these loud sounds. |
|  |  | My plan today was ruined due to the _____ weather! The sky is dark and _____. |
| Positive | Two | On my birthday, my friends brought me so many _____ presents. I love them for being _____. |
|  |  | I had a _____ picnic with my friends. It was a beautiful day, and we had a _____ time together. |
|  |  | My cat made this _____ purr sound that made my heart melt. I love living with this _____ pet. |
|  |  | I hope my _____ friends can come back to visit more often. We had a such a _____ time catching up today. |
|  |  | It was _____ to meet a new classmate today. We had a _____ chat and hung out together. |
|  |  | My teacher said she enjoyed the _____ story I wrote for the writing class. She said it was _____. |
|  |  | I was very engaged with the _____ movie. I also enjoyed some _____ pizza while watching it. |
|  |  | I feel _____ for helping my grandma. She thanked me for my _____ work. |
|  |  | Finishing my work in time is _____. I will go to a _____ party tonight to celebrate. |
|  |  | My family travelled together last week. We enjoyed the _____ mountains and the _____ sunrise. |
| Neutral |  | The cookbook says that "the next step is to place it into a _____ bowl. After you finish, wash the dish with _____ water." |
|  |  | I saw a _____ post office on my way to the library. I walked straight and saw a _____ building on the right. |
|  |  | The _____ button directed me to the next page. I then had to click the _____ box to confirm the booking. |
|  |  | This machine has a _____ mark of the company name in the middle. There is also a _____ button on its cover. |
|  |  | I drove the normal way home, seeing the _____ things as usual. It was just a _____ day anyway. |
|  |  | In the morning, I go to school by hopping on a _____ bus. I always carry my _____ bag with me. |
|  |  | I am at the train station. The ticket says that I should wait at the _____ platform and be ready to get on the _____ train. |
|  |  | I boiled water with the _____ kettle today by putting some water into it. I switched it off after the _____ light flashed. |
|  |  | I went to return a book. I opened the _____ door and put the book on the _____ bookcase. |
|  |  | I do not know who lives in this _____ house. There is a _____ balcony facing the street. |
| Negative |  | I hate these kinds of _____ video games. The _____ scene still makes me shiver. |
|  |  | The _____ principal only scolded me when others were also fighting. I cannot stand this at all. This is so _____. |
|  |  | This accident has caused _____ damage to the cars. It has also resulted in _____ injuries to several people. |
|  |  | I failed at my maths exam again. Am I really _____ at maths? Maybe I am just stupid and _____ at everything. |
|  |  | I fought with my friend today. I feel so _____ and I hope to not have a _____ fight like this again. |
|  |  | The _____ deadline for my homework is just a few days from now. I am so _____ and unprepared. |
|  |  | Being judged by my _____ appearance makes me sad. I cannot choose how I look, even if it is _____. |
|  |  | I fell down the stairs and my legs are _____. I should be more careful next time to avoid this _____ accident. |
|  |  | I want to leave this _____ place at once. It is unbearable. Every second here makes me more _____. |
|  |  | My mood is ruined by the _____ weather. I have lost motivation to work. I hope the _____ weather will be over soon. |

1. **Norming and Validation of Stimuli**

Prior to data collection, the valence of the stimuli was estimated using a *Bidirectional Encoder Representations from Transformers* *(BERT)* model, a transformer-based machine learning technique for natural language processing (Devlin et al., 2019). *BERT* considers the context for each occurrence of a given word to generate word embedding representations for each word. For this validation of stimuli, we directly applied the model *“nlptown/bert-base-multilingual-uncased-sentiment”* from Hugging Face, which was pretrained on product reviews and fine-tuned for sentiment analysis. The output of the model was one of five sentiment classes, from negative to positive. To get a more nuanced sentiment score, we used the *softmax* function to obtain the probability of all five sentiment classes by exponentiating the model’s output logits and normalizing them so that they summed up to 1. From the resulting matrix, we assigned a weight to each sentiment class to obtain the resulting sentiment score for each story. The score was a scalar value that ranges from -1 (very negative) to 1 (very positive). The mean estimated sentiment was -0.41 (*SD* = 0.43) for the narratives in the negative context, -0.12 (*SD* = 0.40) in the neutral context, and 0.77 (*SD* = 0.13) in the positive context. Note that while *BERT* took into account the context to determine sentiment, it was not foolproof and could make wrong predictions. For example, the context about a fight with my friend was rated as 0.70 (very positive) by *BERT*, which deviated from its intended message. Nevertheless, one-way ANOVA showed that the three conditions were significantly different in their sentiment scores, *F*(2, 57) = 62.34, *p* < .001.

To ascertain that the stimuli achieved their intended valence effects, after data collection was completed, we conducted a post hoc analysis of the valence of the stimuli. The independent variable was the pre-determined valence of the narrative context. The dependent variables were the participants judgment of the valence those narratives. The same inclusion criteria apply for the validation study participants, who all reported to be native English speakers based in the UK area, have normal or corrected-to-normal vision, and no history of dyslexia or other language difficulties. They all provided consent before taking part.

***Valence Categorisation***

Twenty participants (18 Females, 2 Males, *M_age_* = 18.39, *SD_age_* = 0.56, range = 18-19) read through the 60 narratives (as in Section 3 above) one at a time. They completed a valence categorisation task after reading each narrative to sort each narrative into either the “positive”, “neutral”, or “negative” valence category. Overall, the mean agreement rate (participants sorting a narrative into the pre-determined category) was 96.75% (*SD* = 17.7%). No item had an agreement rate of less than 80%.

***Valence Rating***

Another group of 19 participants (10 Females, 9 Males, *M_age_* = 25.58, *SD_age_* = 3.40, range = 19-30) did a valence rating task after reading each of the 60 narratives (as in Section 3 above), where they were asked to rate on a Likert scale of 1-7, how positive/negative the narrative made them feel. The descriptive statistics of their ratings (*M* and *SD*) are reported in the table and figure below. Participants’ rating of the valence of the narratives were consistent with their categorisation and *BERT* estimation.

We also calculated a correlation between *BERT* ratings and valence ratings. Pearson correlation shows that *BERT* ratings and valence ratings are strongly positively correlated, *r* = 0.69, *p* < .001.

| Valence | M valence rating | SD valence rating |
| --- | --- | --- |
| Negative | 1.95 | 0.57 |
| Neutral | 4.11 | 0.18 |
| Positive | 5.92 | 0.61 |


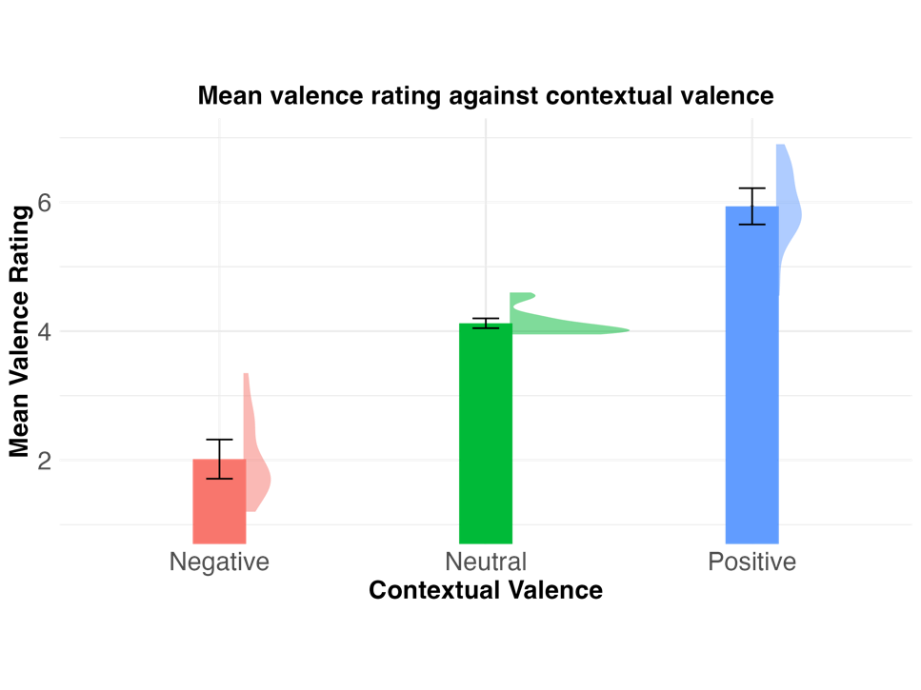


1. **Concreteness of generated meaning**

Following the suggestion of an anonymous reviewer, we also compare the generated word meanings to concreteness ratings (Brysbaert et al., 2014). From a scale of 1-5, with 1 being very abstract and 5 being very concrete, the mean concreteness ratings of children’s generated meanings were 2.66 for the neutral condition (SD = 0.35), 2.56 for the negative condition (SD = 0.38), and 2.52 for the positive condition (SD = 0.37). The values were more abstract than the average concreteness ratings for all words in the lexicon, which was 3.04 (Brysbaert et al., 2014).

1. **Results of exit questionnaires**

At the end of Session 1 participants were asked if they were aware of the study’s aim. Analysis of the results showed that while 31 participants (41%) indicated yes, only 10 of them mentioned the word “context” in their responses and only one participant mentioned emotionality or valence of the context.
